# Cancer metabolites: promising biomarkers for cancer liquid biopsy

**DOI:** 10.1186/s40364-023-00507-3

**Published:** 2023-06-30

**Authors:** Wenxiang Wang, Zhiwei Rong, Guangxi Wang, Yan Hou, Fan Yang, Mantang Qiu

**Affiliations:** 1 Department of Thoracic Surgery, Peking University People’s Hospital, Beijing, 100044 China; 2 Peking University People’s Hospital Thoracic Oncology Institute, Beijing, 100044 China; 3 Department of Epidemiology and Biostatistics, School of Public Health, Peking University Health Science Center, Beijing, 100191 China; 4 Institute of Systems Biomedicine, School of Basic Medical Sciences, Peking-Tsinghua Center for Life Sciences, Peking University Health Science Center, Beijing, 100191 China; 5 Department of Biostatistics, School of Public Health, Peking University, Beijing, 100191 China; 6 Clinical Research Center, Peking University, Beijing, 100191 China

**Keywords:** Circulating metabolites, Cancer diagnosis, Biomarkers, Liquid biopsy

## Abstract

Cancer exerts a multitude of effects on metabolism, including the reprogramming of cellular metabolic pathways and alterations in metabolites that facilitate inappropriate proliferation of cancer cells and adaptation to the tumor microenvironment. There is a growing body of evidence suggesting that aberrant metabolites play pivotal roles in tumorigenesis and metastasis, and have the potential to serve as biomarkers for personalized cancer therapy. Importantly, high-throughput metabolomics detection techniques and machine learning approaches offer tremendous potential for clinical oncology by enabling the identification of cancer-specific metabolites. Emerging research indicates that circulating metabolites have great promise as noninvasive biomarkers for cancer detection. Therefore, this review summarizes reported abnormal cancer-related metabolites in the last decade and highlights the application of metabolomics in liquid biopsy, including detection specimens, technologies, methods, and challenges. The review provides insights into cancer metabolites as a promising tool for clinical applications.

## Introduction

**Fig. 1:**
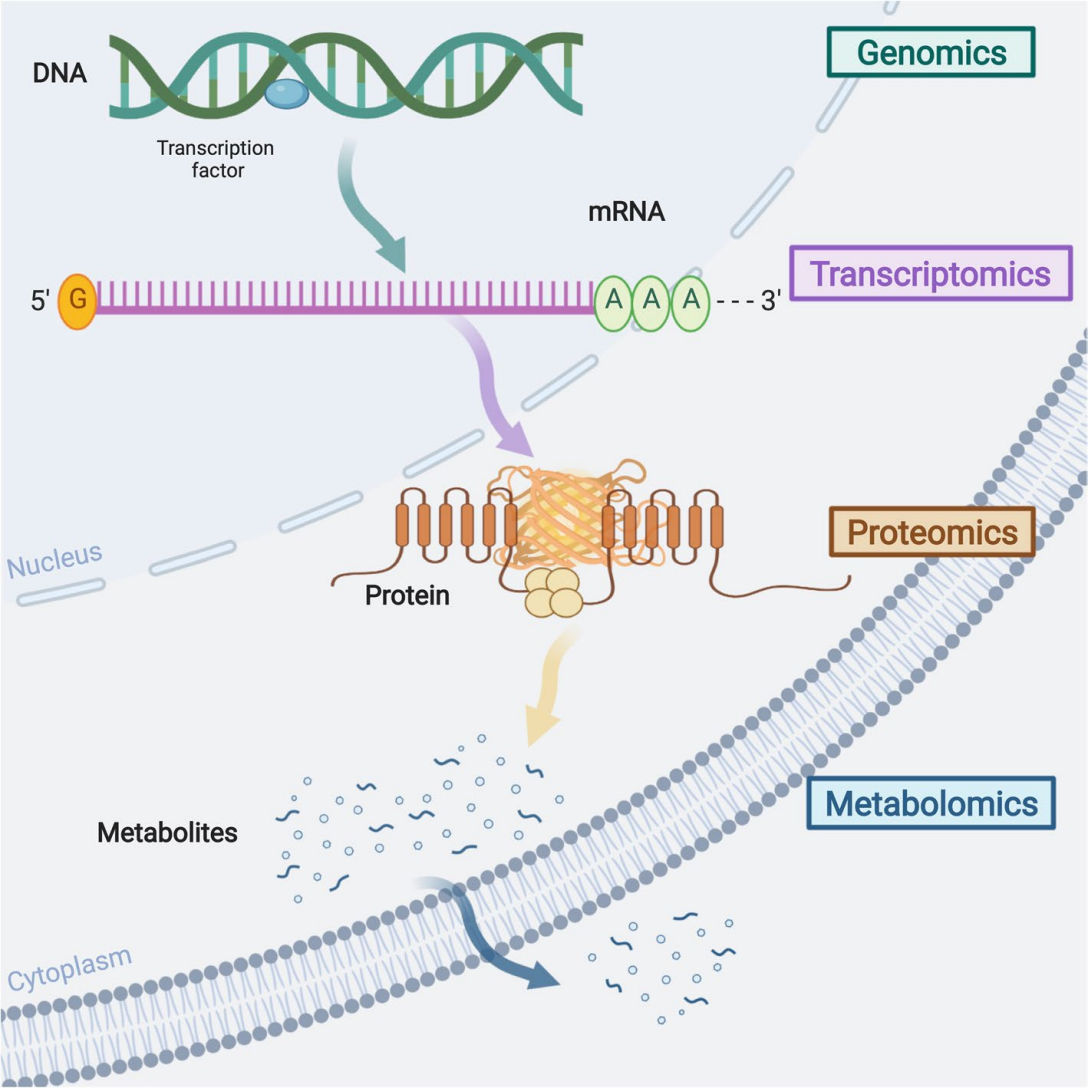
Metabolomics in relation to other omics. Metabolomics, downstream of the genomics, transcriptome and proteomics, can directly reflects the physiological and pathological conditions of an individual

To date, cancer remains one of the leading cause of death in over 100 countries. According to the global cancer statistics in 2021, the morbidity and mortality rates of cancer rates of cancer continue to rise worldwide, with 19.29 million new cases and 9.96 million cancer-related deaths [1]. These alarming statistics emphasize the need to focus on the search for promising cancer biomarkers. Metabolomics, a novel and promising research tool, involves the systematic identification and quantification of all metabolites in a given organism or biological sample for exploring the relationship between metabolites and disease including cancer [2, 3]. Importantly, the metabolome is downstream of the genome and proteome networks, directly reflecting an individual's physiological and pathological state (Fig. 1). All features support the application of metabolomics as a powerful tool for identifying cancer biomarkers and understanding the drivers of tumorigenesis [4, 5].

Dysregulated cellular metabolism is the significant hallmarkers of cancer [6]. The metabolic reprogramming of cancer cells, which supports uncontrolled proliferation, leads to changes in normal metabolite levels and the production of abnormal metabolites. These altered metabolites, collectively known as aberrant metabolites, are the end products of biological metabolism and exhibit high sensitivity to biological activity and pathological conditions [7]. As a result, these aberrant metabolites, which reflect specific metabolic phenotypes and tumor activity, can serve as biomarkers for early cancer diagnosis [8], high-risk patient identification [9], and treatment response assessment [10]. The altered metabolism in cancer cells also creates dependencies or resistance to specific metabolites, which can be targeted with precision medicine in certain casesAltered metabolism also results in unique metabolites dependence or resistance, which in some cases can be targeted with precision medicine [11]. Technically, metabolomics enables the collection, detection, and analysis of various types of cancer-related metabolites. Therefore, the detection of abnormal cancer metabolites through metabolomics offers promising opportunities for the development and discovery of tumor biomarkers.

In recent decades, a significant body of evidence has emerged indicating that cancer metabolites have the potential to serve as biomarkers for the pathogenesis and development of cancer [12]. In this review, we summarize existing literature on metabolites in the diagnosis and prognosis of cancer, and present the current progress and challenges in the field of metabolomics, with the intention of yielding further insights into the potential role of cancer metabolites in clinical application.

## Aberrant metabolites could be biomarkers for cancer detection

As we are aware, aberrant metabolites can be utilized for cancer detection, disease staging [13], risk stratification [9], and therapeutic response monitoring [14] and so on. In the condition of nutritional deficiency, differential metabolites not only act as cell intrinsic signaling regulators for cancer phenotype and redox homeostasis [15], but are also secreted into extracellular matrix, coordinating intracellular activity and tumour environment that supports malignancy [16]. In turn, tumor cells can alter metabolic fluxes and reprogram their metabolism to maintain their own biosynthesis and energy requirements within oncogenic mutations and tumour suppressor contexts [17, 18]. As metabolites have such a tremendous impact on cancer initation and evolution, we aim to categorize cancer metabolites referred to in the current literature and investigate the clinical value of aberrant metabolites as potential biomarkers.

### Metabolites involved in glucose metabolism

Since Otto Warburg's discovery in the 1920s that tumor cells preferentially metabolize glucose into lactate despite abundant oxygen availability [19], researchers have increasingly recognized the significance of glucose metabolism in cancer.

Oncometabolites, which mainly include 2-hydroxyglutarate, succinate and fumarate, refer to metabolites that drive distinct cancers and arise in human cancer due to somatic mutations in the isocitrate dehydrogenase (IDH) genes, fumarate hydratase (FH), or succinate dehydrogenase (SDH) gene [20]. An increasing number of studies suggest that these intermediary metabolites act as signaling molecules that promote tumor growth by inhibiting epigenetic enzymes and suppressing DNA repair [21]. Of note, tissue oncometabolites have been shown to correlate with progression-free and overall survival of cancer patients [22]. Hence, oncometabolites are expected to be diagnostic biomarkers for cancer. Further investigations towards oncometabolites may guide the development of effective strategies for targeted therapy that could diminish tumorigenesis.

Pyruvate is the pivotal junction between oxidative phosphorylation and glycolysis, regulated by many enzymes and mitochondrial transporers which together control carbon flux. Pyruvate Kinase (PK), particularly PKM2, is viewed as a desirable target because its enzymatic activation enhances glycolysis and promotes tumorigenesis [23]. Lactic acid, the primary metabolic product of anaerobic glycolysis, is linked to oxidative stress resistance and lipid biosynthesis in cancer cells. Recent studies have shown that lactate enhances ferroptosis resistance in hepatocellular carcinoma (HCC) cells, contributing to tumor growth [24]. What’s more, lactic acid accumulated during cancer metabolism serves as a precursor for histone lysine lactation modification, which regulates gene expression [25]. Additionally, stromal-derived lactate promotes the accumulation of lipid droplets [26] and extracellular acidification of tumor microenvironment [27] to foster cancer metastatic.

### Metabolites involved in lipid metabolism

Lipids are essential for signal transduction, energy metabolism and membrane structural integrity. However, excessive levels of circulating lipids have been linked to cancer malignant progression through “lipotoxicity”, which results in oxidative stress, mitochondrial dysfunction, and impaired autophagy [28].

Free Fatty acid (FFA), which are the building blocks of all lipids, can be categorized as either unsaturated or saturated. On the one hand, numerous researches have demonstrated that serum polyunsaturated fatty acids possess great sensitivity and specificity for cancer early detection [29]. For example, a multi-omics investigation has identified plasma arachidonic acid (AA) and linoleic acid (LA) as potential biomarkers for non-small-cell lung cancer (NSCLC) clinical diagnosis due to their association with cancer progression through Akt pathway [30]. Furthermore, oxylipins, metabolites of polyunsaturated fatty acids, have been differentially expressed in early-stage breast cancer, suggesting a new approach for cancer detection [31]. On the other hand, saturated FFAs and esterified fatty acids (EFA) can be used to identify patients with epithelial ovarian cancer (EOC) based on the metabolomics approach [32].

Cholesterol plays a critical role in regulating cell structure and substance metabolism by acting directly or indirectly as signaling molecules [33]. For instance, oxysterol 27-hydroxycholesterol (27HC) targets liver X receptor (LXR) and contributes to breast cancer progression [34]. Furthermore, the metabolic reprogramming of cholesterol also regulates tumor microenvironment by indirectly affecting the biological behavior of immune cells. A study by Ma X., et indicated that cholesterol in the tumor microenvironment induces immune checkpoint expression and exhaustion in CD8 + T cells [35]. Recently, a large number of studies have revealed that targeting abnormal cholesterol metabolism can be a novel diagnostic and therapeutic approach [36, 37].

Phospholipids are currently implicated in the pathophysiology and progression of several cancers, although the mechanisms underlying their impact on cancer biology are still emerging. According to the metabolic database from different cohorts for various cancers at multiple research centers, phospholipid reprogramming is considered closely relevant to cancer diagnosis and prognosis (Table 1).

**Table 1:** Phospholipids for cancer diagnosis and prognosis

Author	Year	Cancer Type	Sample Type	Method	Lipid metabolites	Effect	Ref
His, M., et al.	2019	Breast cancer	Blood	MS, targeted	PC ae C36:3, PC aa C36:3, PC ae C34:2, PC ae C36:2, PC ae C38:2	Breast cancer risk prediction	[38]
Han, X., et al.	2020	Lung cancer	Blood	UPLC-MS, targeted	Cer 36:1–3, Cer 38:1–3, SM 36:1–2, LPC 16:1	EGFR-TKI therapeutic efficacy evaluation	[39]
Rohnisch, H. E., et al.	2020	Prostate cancer	Blood	MS and NMR, targeted	LPC C17:0, LPC C20:3, LPC C20:4, PC ae C38:3, PC ae C38:4, PC ae C40:2	Prostate cancer risk assessment	[40]
Jiang, N., et al.	2021	Papillary Thyroid Cancer	Blood	UPLC-MS,untargeted	GlcCer(d14:1/24:1), PE(18:1/18:1), SM(d16:1/24:1), SM(d18:1/15:0), SM(d18):1/16:1)	Papillary Thyroid Cancer Diagnosis	[41]
Wang, G., et al.	2021	Pancreatic ductal adenocarcinoma	Blood	LC–MS, untargeted and targeted	DG: 18:1–18:1; LPCs: 14:0, 16:0, 18:1, 20:4; PCs: 16:0–16:0, 16:0–18:1, 18:0–18:2, 18:0–20:3, 16:0–22:5, 18:0–22:5, and O-16:0–18:2; LPE: 22:4; PE: 16:0–18:2; SM: d18:1/18:0, d18:2/24:1, d18:2/24:2	PDAC detection	[42]
Wang, G., et al.	2022	Lung cancer	Blood	LC–MS, untargeted and targeted	PC16:0–18:1, PC 16:0–18:2, PC 18:0–18:1, PC 18:0–18:2, PC 16:0–22:6	Early-stage lung cancer detection	[43]

*PC* phosphatidylcholines, *Cer* Ceramide, *SM* sphingomyelin, *LPC* Lyso Phosphatidyl choline, *PE* Phosphatidyl ethanolamine, *LPE* Lyso Phosphatidyl ethanolamine, *DG* diacylglycerol, *MS* mass spectrometry, *UPLC* ultra-high performance liquid chromatography, *NMR* nuclear magnetic resonance

### Metabolites involved in Amino acid metabolism

Tumor cells require an adequate supply of amino acids to support their proliferation, survival, and biosynthesis. Multiple amino acids have pleiotropic effects on tumor evolution, and targeting amino acid metabolism could benefit potential cancer therapies.

Serine serves as the precursor for the amino acids glycine and cysteine, purine nucleotides, and glutathione. It regulates one-carbon metabolism by supplying a one-carbon unitt [44]. Some tumors essentially hyperactivate intracellular serine de novo synthesis by utilizing glycolytic intermediate metabolites [45]. Differential expression levels of serine metabolism-related proteins and enzymes in several cancer subtypes are significantly correlated with clinical prognostic outcomes [46]. Of course, targting serine metabolism would provide a new treatment idea for cancer.

Glutamine, the most studied amino acid, is highly expressed in cancer cells and associated with overall survival and drug resistance in the majority of cancer types [47]. Some metabolic molecules and axes ultimately promote or inhibit cancer malignant progression by regulating glutamine metabolism [48, 49]. So far, several perspective treatments deploy strategies targeting glutamine metabolism, showing great potential for clinical applications [10, 50].

Asparagine coupled with aspartate, exert an influence on cancer evolution and metastasis by sustain NAD + /NADH homeostasis [51]. Several researchers have proposed that asparagine from cervicovaginal fluid could be used for detecting endometrial cancer [52]. Enhanced tryptophan metabolism has been reported in multiple tumor types and can be coverted into many biologically active substances such as kynurenine (Kyn) and serotonin (5-HT). Growing evidence suggests that tryptophan and its metabolites can be used as biomarkers for cancer risk [53]. Latest studies have found that the Interleukin-4-induced-1 (IL4I1) enzyme can also catalyse tryptophan to generate kynurenine and indole metabolites, complementing the tryptophan metabolic pathway and opening up new avenues for cancer treatment [54].

Branched-chain amino acids (BCAAs), including leucine, isoleucine, and valine are essential for cancer cell growth by activating the mechanistic target of rapamycin complex 1 (mTORC1) and supplying carbon sources for energy production. Similarly, BCAAs have been observed to discriminate patients with cancer and benign disease as valuable biomarkers [55]. Methionine, including S-adenosyl methionine (SAM), is linked to one-carbon metabolism and methylation status, thereby regulating chromatin accessibility and gene transcription. As early as 2016, Palanichamy K, et al. proposed that methionine activates oncogenic kinases in glioblastoma to promote cancer cell proliferation [56].

All in all, understanding amino acid metabolism has become a new entry point for comprehending cancer mechanisms and developing treatment methods.

### Metabolites involved in nucleotide metabolism

As biological information macromolecules, nucleotides primarily function as the raw materials for nucleic acid synthesis to support cell proliferation. As we know, proliferating cancer cells tend to synthesize nucleotides de novo [57], and different tumor subtypes possess distinguishing nucleotide reprogramming procedures [58]. In recent decades, considerable attention has been focused on the role of nucleotide metabolism in cancer development.

In the process of tumorigenesis, an imbalance in purine metabolism and upregulation of relative enzymes provide cancer cells with necessary energy and cofactors, thereby promoting tumor formation and growth. The Austrian Academy Research Center found that cellular purine supply and synthesis can affect the activity of the chromosome modifying protein BRD4, thus affecting chromatin accessibility and promoting carcinogenesis [59]. Moreover, the roles of N6-methyladenosine (m6A) modifications in the cancer progression and immunotherapy have been summarized.

Meanwhile, pyrimidine nucleotides metabolism also plays a significant role in the onset and progression of cancer. Aarif et al. found that thymidylate synthase (TS) gene was associated with epithelial-mesenchymal transition (EMT), and TS-deficient cancer cells showed decreased invasion and metastasis [60]. By analyzing public databases on ovarian cancer, investigators found that distinct 5-Methylcytosine (m5C) modification patterns exhibited metabolism heterogeneity, which consequently led to survival differences, providing evidence for cancer risk stratification [61]. Uridine and pseudouridine also play a critical role in nucleoside synthesis and reduction of cytotoxicity, representing meaningful risk factors for cancer [62].

### Other metabolites

Vitamins have been related to the development of various cancers and may be promising agents in cancer treatment. Take an example of nicotinamide (NAM), a water-soluble amide form of niacin (vitamin B3), not only increases lipid metabolism and energy disruption in breast cancer [63], but the related NAD + metabolism can induce PD-L1 expression to drive tumor immune escape [64]. This provides insight into NAM supplements as next-generation antimetabolites for cancer treatment.

Metabolites derived from gut microbiome dysbiosis contribute to tumorigenesis by inducing inflammatory factors and oxidative DNA damage. Chen et al. have reported 332 gut microbiome-associated serum metabolites (GMSM) were significantly altered in colorectal cancer (CRC) and adenomas through microbiome-metabolome integration analysis. They also developed a novel panel composed of eight GMSM that was qualitatively and accurately evaluated by targeted metabolomics and validation cohort, with an AUC of 0.92, a sensitivity of 83.5%, and a specificity of 84.9%, which can serve as predictor for CRC and colorectal adenomas [65].

Exosomes, as biological messengers of intercellular communication system, mirror cellular features and physiological states by means of secreted vesicle contents [66]. A study by Zhang, C., et al. illustrated that colorectal cancer cell-derived exosomal could promote pre-metastatic niche formation and liver metastasis via reprogramming lipid metabolism in cancer-associated fibroblasts, which may be a potential therapeutic target [67].

Overall, cancer metabolites regulate a variety of cellular activities, including oncogenic signal, tumor-niche interaction, intratumor heterogeneity and immune availability. Defining potential metabolites biomarkers and exploring the mechanism behind metabolic scenarios are crucial mandates to guide clinicians in cancer management and treatment.

## Cancer metabolomics: specimens, techniques, methodologies and analysis

Metabolomics refers to the analysis of small molecule metabolites (≤ 1500 Daltons and nonpeptide) in biological specimen [12]. Cancer metabolomics can be qualitatively and quantitatively employed to analyze various sample sources, which include tumor tissues, bio-fluids, cancer cells and microorganisms [68]. And the selection of samples is also dependent on the origin and primary location of the cancer [69], such as serum/plasma for solid tumor, exhaled breath for lung cancer, the saliva for oral squamous cell carcinoma [70], urine for bladder cancer [71], and aqueous humor for retinoblastoma [72]. The most commonly used type of samples for cancer research are peripheral blood, due to its easy availability and the routine of blood sampling in clinical scenario. Given that metabolic profiles are susceptible to many factors, the collection procedure of samples need to be strictly controlled according to the experimental design and sample characteristics, and consider the parallelism of sample collection time, preservation conditions and preservation time [73]. All samples collected should be extracted immediately in order to inactivate enzymes in tissues or cells and prevent metabolites from degrading, usually by liquid nitrogen quick-freezing method [74]. Different biological samples have different characteristics and are suitable for different research purposes. In particular, mass spectrometry imaging (MSI) technology allows the direct visualization of metabolite distributions in tissues samples, thus enabling the discovery of key biomarkers with cancer diagnostics potential [75]. In view of the accessibility of tumor tissue samples in human and animal models, advanced three-dimensional cell models have proven to be capable of depicting architectural and microenvironmental features of several tissues, contributing to a better understanding of disease development, pathology onset and progression mechanisms [76].

The core of metabolomics is the techniques for extensive detection and identification of metabolites and the efficiency for accurate qualitative and quantitative them. Nuclear magnetic resonance (NMR) and mass spectrometry (MS) are still cornerstones for metabolomic analysis [77]. NMR technology, especially 1H NMR, is a spectroscopic technology used for metabolic fingerprint research and in vivo studies [78]. Now, 2DNMR is the most general and versatile method in complex mixture analysis and is widely used in many fields and researches [79]. MS routinely combined with chromatographic separation phase are divided into three types including capillary electrophoresis-mass (CE-MS), gas chromatography mass (GC–MS), and liquid chromatography-mass (LC–MS), thus providing highly specific analysis and exactly chemical information [68]. But there are still several problems in MS-based metabolomics, such as complex sample preparation and low reproducibility, which actuates the advancement in novel analytical methods and the reformation of existing technologies. Taking ion migration spectrometry combined with mass spectrometry (IMS-MS) as an example, peak overlap is avoided the coverage of metabolome is expanded [80]. In addition, the strategy of simultaneous acquisition of MS1 and MS2 spectra can improve the accuracy of the identification of metabolic biomarkers [81]. Moreover, Metabolomics may be divided into data-dependent acquisition (DDA) mode–based untargeted metabolomics and multiple reaction monitoring (MRM) mode–based targeted metabolomics, depending on the detection strategy [82]. Targeted metabolic profiling approach is capable of identifying metabolite marker candidates with high sensitivity and reproducibility and a wider linear range. However, this targeted method is a biased analysis because it requires an advanced knowledge background and focuses only on some specific metabolites [83]. To expand the metabolome coverage and articulate a better panel of metabolites, non-targeted metabolomics are often used to screen for undeviated and comprehensive systemic metabolome characteristics and select metabolites subset for further targeted detection [84]. Because both of targeted and non-targeted metabolomics have their advantages and disadvantages, the two metabolomics methods are often used in combination and play a role together in practical applications [85].

Due to the chemical complexity and dynamic changes of metabolites, the current metabolomics still has some shortcomings, such as limited metabolites coverage, insufficient detective sensitivity, low qualitative and quantitative accuracy, and lack of spatial information. On account of these limitations, more and more metabolomics methods and instruments are being improved or further improved [12]. In order to improve the qualitative and quantitative accuracy of non-targeted metabolomics, global untargeted metabolomics detection technology is introduced to achieve higher validation efficiency and lower false negative rate by isotope internal standard of known concentration and multiple dilution of QC samples [86]. Studies have performed global untargeted metabolomics profiling in urine of African American and white smokers to characterize the pattern of metabolites, identify differentially regulated pathways, and correlate these profiles with lung cancer risk [87]. Meanwhile, targeted metabolism using the 3-nitrohydrazine derivatization strategy greatly improves the coverage of metabolites in a single assay, and significantly improves the detection sensitivity of metabolites [88]. It has been demonstrated that 324 metabolites can be identified rapidly and quantitatively by automated high-throughput metabolite array technology, including fatty acids, amino acids, organic acids, carbohydrates, and bile acids [89]. Furthermore, spatial metabolomics integrated mass spectrometry conducts qualitative, quantitative and positioning accurate analysis of thousands of metabolites in tissues, providing new visual perspective for tumor biomarkers screening, edge differentiation and pharmacokinetics [90]. Our study combined with matrixassisted laser desorption/ionization MS imaging (MALDI-MSI) further clarified the expression of feature lipids in lung cancer tissues in situ and enhanced the potential and credibility of lipid biomarkers [43]. In addition, it is also demonstrated that the distribution of carnitine in breast cancer tumor tissues was heterogeneous based on same metabolic technology, with the highest content in the central region and the lowest content in the distal normal region [91]. With the assistance of this technology, a metabolic classifier with high accuracy was developed for the study of NSCLC patients who received chemotherapy and those who did not receive, providing another method for evaluating the histopathological response of NSCLC patients [92].

The above are all about metabolites detection, which provide solid foundation for metabololomics data analysis. How to find significant abrrent metabolites in high-throughput metabolic data is the difficult and important point of metabolomics, requiring more statistical methods and further exploration. Based on the strategy of modeling interactions, the differential metabolites detection methods can be classified into two categories: univariate and multivariate statistical methods [93]. Univariate methods analyze metabolomics features independently and easily, including Student’s t-test, Analysis of variance (ANOVA), Mann–Whitney U test, or Kruskal–Wallis one-way analysis of variance. In the process,it is important to find a statistically significant result by chance (i.e., false positive rate). In order to control the false positive rate, several correction methods are available. The most conservative (less false positives and more false negatives) approach is the Bonferroni correction, where the significance level for one hypothesis (i.e., alpha value) is divided by the number of hypotheses tested simultaneously [94]. Other less conservative methods are mostly based on the minimization of the false-discovery rate (FDR [95];), which have been extensively used for parallel analysis of data from thousands of gene expression microarrays [96].

In contrast to univariate methods,, multivariate analyses involving supervised and unsupervised methods, take into account all the metabolomic features simultaneously and consequently and identify relationship patterns between them. Unsupervised methods provide an effective way to detect data patterns that are correlated with experimental and/or biological for complex metabolomic data. Principal component analysis (PCA) is the most commonly used unsupervised method in metabolomic studies [97]. Through orthogonal linear transformation, a group of metabolic characteristics is converted into a group of uncorrelated variables, so as to achieve dimensionality reduction analysis of high-dimensional metabonomics data. Recently, t-Distributed Stochastic Neighbor Embedding (t-SNE) can converts similarities between data points to joint probabilities and tries to minimize the Kullback–Leibler divergence between the joint probabilities of the low-dimensional embedding and the high-dimensional data in metabolomics studies [98].

Supervision method can better identify metabolic patterns and related phenotypic variable by reducing the weight of other sources of variance. Partial least squares (PLS [99];) and revised orthogonal PLS (O-PLS [100];) models not only correlate with the variable of interest and a second uncorrelated component, but also provide Variable Importance in Projection (VIP), allowing for evaluating importance of individual variables from the predictors block influence and easing the difficulty of differential metabolites selection. Furthermore, some supervised machine learning methods have been applied in metabolomics studies. Support vector machines (SVMs), random forests (RF), or deep neural networks (DNNs) are other supervised analysis methods to build classifiers based on metabolomics data [101, 102].

With the ongoing metabolomics researches, it is urgent to improve the comparability of metabolomics measurements by standardizing methods and results. Since 2005, the Metabolomics Standards Initiative has been formed [103]. And suitable reference materials (RMs) have been developed serving for quality assurance and quality control (QA/QC) in differential metabolomic studies, interlaboratory comparisons, laboratory and instrument qualification [104]. For the most common mass spectroscope-based metabolomics, Alseekh, S et al. proposed a guideline covering sample preparation, replication and randomization, quantification, recovery and recombination, ion suppression and peak misidentification, as a means of achieving high quality reporting of metabolomic data [105]. Morever, in clinical and epidemiological studies, largescale and multisite subjects has narrowed metabolomics bias and created confidence in analytical performance. Although there is no standardized research method for metabolomics at present, the standardization, comparability, repeatability and reproducibility of metabolome can be achieved to the greatest extent through strict operation flow and standardized analysis procedures.

Metabolomics is a static study which characterizes the abundance of a large scale of metabolites in various samples at a certain time, while metabolic fluxomics further tracks the metabolic activities in a dynamic manner through isotope labeling [106]. Many tracers have been used to measure specific metabolic activities. Stable metabolic flux analysis (MFA) can quantify the flux rates by measuring downstream isotope tracing-based metabolites at multiple time points [107]. Recent advances in MFA technologies make them powerful tools for characterizing and quantifying and metabolic activities in cancer research. For instance, U-^13^C] labeling analysis showed that pyruvate metabolism and fatty acid oxidation were significantly enhanced in lung cancer resistant cells after treatment with trametinib, which synergically provided power for OXPHOS system [108].

## Circulating metabolites: an emerging paradigm for liquid biopsy of cancer

Compared to other liquid biopsy markers, circulating metabolites can instantly reflect organic whole status and human biological and pathologic activities, serving as potential diagnostic biomarkers of cancerous clinical utility. Besides, specific metabolites panel are increasingly being taken seriously because of their potential on core areas of oncology, including diagnosis (early screening and detection) and prognosis (postoperative treatment and survival).

On the one hand, early screening and diagnosis has always been the focus of cancer reaserch and the potential application field of cancer metabolomics. At present, selected specific metabolites by machine learning or statistics analysis have effectively and accurately distinguished healthy people from cancer patients [42, 43], and the identification of benign and malignant suspicious nodules is still being further explored [109]. In addition, some studies based on serum metabolomics have highlighted different specific biomarkers in the application of high-risk population identification [110] and early and advanced carcinoma differences [111]. On the other hand, an increasing amount of evidence has proven that circulating metabolites can instantly reflect therapy efficency and cancer recurrence. Combined plasma panel biomarker used for cancer treatment is quite convenient to implement in clinical practice [112, 113]. Using metabolomics method, specific metabolits have been characterized and possess a great capability to monitor the progression and metastasis of cancer [114, 115]. (Table 2).

**Table 2:** Evidence on the circulating metabolites as diagnostic biomarkers in cancers

Application	Authors	Year	Cancer Type	Methodology	Findings(Ref)
Dignosis	Yu, S., et al	2022	Papillary thyroid cancer	UHPLC-MS	A novel metabolic biomarker signature was identified to discriminate papillary thyroid cancer from the benign thyroid nodule [109]
	Wang M., et al	2022	Colorectal cancer	UPLC-TOF–MS	By screening the differential plasma metabolites and further quantitative analysis, we found the plasma biomarkers that can be used in the diagnosis of colorectal cancer. [110]
	Ossoliński K., et al	2022	Bladder cancer	NMR, LDI-MS, ICP-OES	Three different analytical platforms demonstrate that the identified distinct serum metabolites have potential to be used for noninvasive detection, staging, and grading of BC [116]
	Wang G., et al	2022	Lung cancer	UHPLC-MS	﻿A machine learning model made of nine lipids, named Lung Cancer ﻿Artificial Intelligence Detector, effectively identifies patients in the early stages of lung cancer [43]
	Wang G., et al	2021	Pancreatic ductal adenocarcinoma cancer	UHPLC-MS	They optimized 17 characteristic metabolites as detection features and developed a liquid chromatography-mass spectrometry-based targeted assay, proposeing that the machine learning-aided lipidomics approach be used for early detection of PDAC [42]
	Casadei-Gardini A., et al	2020	Hepatocellular Carcinoma	NMR	This study analysis identified a set of metabolites with possible clinical and biological implication in HCC pathophysiology [111]
Prognosis	Triozzi, P. L., et al	2022	Melanoma	UPLC-MS	Blood metabolomics as predictive biomarkers reflect patient response to anti-PD-1 immune checkpoint therapy [112]
	Liu, L., et al	2022	Esophageal squamous cell carcinoma	GC-TOFMS	A panel of 12 esophageal squamous cell carcinoma tumor-associated serum metabolites has the potential for monitoring surgery efficacy and disease relapse [114]
	Zhuang J., et al	2022	Bladder cancer	NMR, UPLC-MS	Serum metabolic profiles of neoadjuvant chemotherapy sensitivity are significantly different in bladder cancer patients. Glycine, hypoxanthine, taurine and glutamine may be the potential biomarkers for clinical treatment [113]
	Luo X., et al	2020	Pancreatic cancer	UPLC-MS	Five new metabolite biomarkers in plasma were verified and can be used to diagnose pancreatic cancer. And Succinic acid and gluconic acid have strong ability to monitor the progression and metastasis of pancreatic cancer [115]
Combination	Huang Y., et al	2022	Breast cnacer	NPELDI-MS	It provide an efficient serum metabolic tool to characterize breast cancer and highlight certain metabolic signatures as potential diagnostic and prognostic factors of diseases. [117]

*UHPLC/UPLC* Ultra-High Performance Liquid Chromatography, *MS* Mass spectrometry, *GC* gas chromatography, *TOF–MS* Time-of-flight mass spectrometry, *NMR* nuclear magnetic resonance, *LDI-MS* laser desorption/ionization mass spectrometry, *ICP-OES* inductively coupled plasma optical emission spectrometry, *NPELDI-MS* nanoparticle-enhanced laser desorption/ionization mass spectrometry PD-1 programmed cell death protein 1, *PDAC* Pancreatic ductal adenocarcinoma cancer, *BC* bladder cancer, *HCC* Hepatocellular Carcinoma

Cancer metabolism is often used to explore a common set of altered metabolites that accompany malignancy, but in reality tumors are metabolically heterogeneous. A meta-analysis aggregating metabolomics data from over 100 different cohorts covering 18 tumor types revealed only a small number of features were mutual across multiple cancers, while many metabolic features varied among different cancers﻿ [118]. Blood-based NMR metabolomics achieved 95% cancer detection rate among 304 recruited participants with nonspecific cancer symptoms, verifying the potential of metabonomics in clinical oncology [119]. Considering the differences and specificities in various cancers, most current studies mainly focused on single cancer types [42, 43]. With the advancement of high-throughput detective technologies, high speed, accurate, and reliable circulating metabolites hold promise for the clinical translational medicine, including cancer diagnostics and prognosis. How to find more tumor-specific metabolites has always been one of the most challenging directions in the field of cancer research.

However, there are still some challenges that need to be addressed in the translation of circulating metabolomics into clinical application. One of the main challenges is the difficulty in interpreting the relationship between metabolic biomarkers and disease. Although there are mature processse for metabolomics data extraction and analysis, and several commonly used databases, many of the metabolites or biomarkers identified in metabolomics research are yet to be confirmed [120, 121]. Compared with other liquid biopsy methods, such as CTCs and ctDNA (Table 3), the plasma metabolome is poorly specific and interpretable and does not reflect the progression and development of cancer subtypes. Furthermore, the relationship of most newly found biomarkers with the development of the disease is yet to be determined and requires further investigation and multi-omics data support. While circulating metabolomics faces several challenges, recent findings have highlighted its potential in cancer diagnostics, representing a new approach for cancer liquid biopsy. We believe that future research focusing on high throughput detection of tumor-specific circulating metabolites will reveal cancer metabolic profiles and provide insights for developing therapeutic targets.

**Table 3:** Comparision of several common liquid biopsy methods

Biomarkers	CTCs	ctDNA	Exosomes	Metabolites
Sample requirement	5-10 ml	15-20 ml	> 5 ml	~ 100ul
Sample preparation	No need	Need, easy	Need, difficult	Need, easy
Detection content	Tumor cells, DNA mutations	Mutations, methylation, fragmentomic	miRNAs, lncRNAs, circRNAs, protein	Small molecular metabolites
Cost	High	High	High	Low
Repeatability	Low	Med	Low	High
Interpretability	High	High	High	Medium
Operability	Difficult	Med	Very difficult	Easy
Stability	Low	Medium	Medium	Low
Application	Diagnosis, prognosis, therapeutic monitoring	Diagnosis, prognosis, therapeutic monitoring	Diagnosis, therapeutic monitoring	Diagnosis

*CTC* circulating tumor cell, *ctDNA* circulating tumor DNA, *NGS* Next-generation sequencing, *miRNA* micro RNA, *mRNA* messenger RNA

## Perspectives and future directions

The development of chromatographic separation and mass spectrometry, as well as advances in molecular biology, has sparked increased interest in liquid biopsies as an early detection tool for cancers, including blood metabolites. Circulating metabolites offer a minimally invasive method to diagnose cancers and hold enormous potential for clinical application. However, many obstacles need to be overcome before cancer metabolites can be widely used.

Firstly, metabolite qualitation and quantitation currently lack uniform methodologies. It has been reported that targeted and untargeted metabolic assays differ in qualitative and quantitative principles [122]. Non-targeted metabolomics identifies substances by primary and secondary spectral information and obtains the relative quantification of the substance. Vice versa, the targeted metabolomics confirms metabolites by combining dual information of precursor and product ions and achieve absolute quantification by establishing the standard substance curve. In the substance discovery phase, untargeted metabolomics analysis is often employed, but the lack of internal standards means only the information of metabolites in the public and in-house database is relied upon. Furthermore,, the detection performance also vary across different instrument platforms. Therefore, it is urgently necessary to develop sensitive and reproducible methods for detecting serum metabolites.

Secondly, metabolites heterogeneity exists among different cancers. Metabolic profiles of tumors are dependent on the genotype and tissue of origin [123]. Metabolic reprogramming often occurs in various human cancers, driven by oncogene and tumor suppressed genes. A study by Priolo, C., et al. demonstrated that selected metabolites are differentially accumulated in the MYC-high versus AKT1-high tumors. The former was associated with dysregulated lipid metabolism, whereas the latter was related to the accumulation of aerobic glycolysis metabolites [124]. Besides, cancer stem cells (CSCs), a unipotent cell population present within the tumor cell mass, exhibit distinct metabolic properties and reprogramming characteristics which contribute to tumor metastasis and therapy resistance [125, 126].

Lastly, circulating metabolites are influenced by multiple factors. It is well known that serum metabolome is affected by exogenous factors such as environmental and dietary factors, as well as endogenous factors such as DNA/mRNA/protein. The origins of circulating metabolites are complicated and include highly heritable metabolic products or metabolites influenced by the gut microbiome, lifestyle choices (such as smoking), and diet [127]. Furthermore, it has been challenging to determine the extent to which individual factors directly impact systemic metabolic status. Therefore, it is required to fully understand the key determinants of metabolites. To be more specific, metabolism studies should be conducted with participants whose overall metabolic status is similar and realistic, including age and sex matching, dietary control, nutritional assessment, and medication records.

To address these challenges, many studies have begun to combine multi-omics with metabolomics to further explore the mechanism behind disease occurrence and development. Metabolomics plus genomics can integrate gene expression differences and aberrant metabolites changes to study the biological changes of disease from the "cause" and "effect" levels. This approach can help discover more effective and accurate cancer biomarkers. For example, Zhou, L., et al. provide new insights into metabolism-related gene landscape in order to predict prostate cancer recurrence and treatment response [128]. Metabolomics combined with proteomics can systematically describe the regulatory process from protein to metabolites and explore the upstream and downstream regulatory pathways of crucial biomarkers. A recent study delineates the copper-metabolic-metastasis axis of high-risk triple negative breast cancer by proteomic and metabolomic approaches, providing a theoretical basis for the next generation of cancer therapies [129]. In addition, matching metabolome with the latest microbiome by means of metagenomic sequencing or 16S sequencing can reveal the important role of intestinal microbiome-related metabolites in cancers [65].

Overall, high-throughput sequencing has transformed biological processes into data, which can help to understand the underlying mechanisms of biological systems. Through the integration of deep learning models and the combinational analysis of multiomics data, the conceptual biological regulatory network centered on metabolomics is constructed for exploring new potential biomarkers and therapeutic targets. Understanding the metabolic characteristic changes at the transcriptional and protein levels would help us to identify new biomarkers and novel therapeutic targets.

## Conclusions

**Fig. 2:**
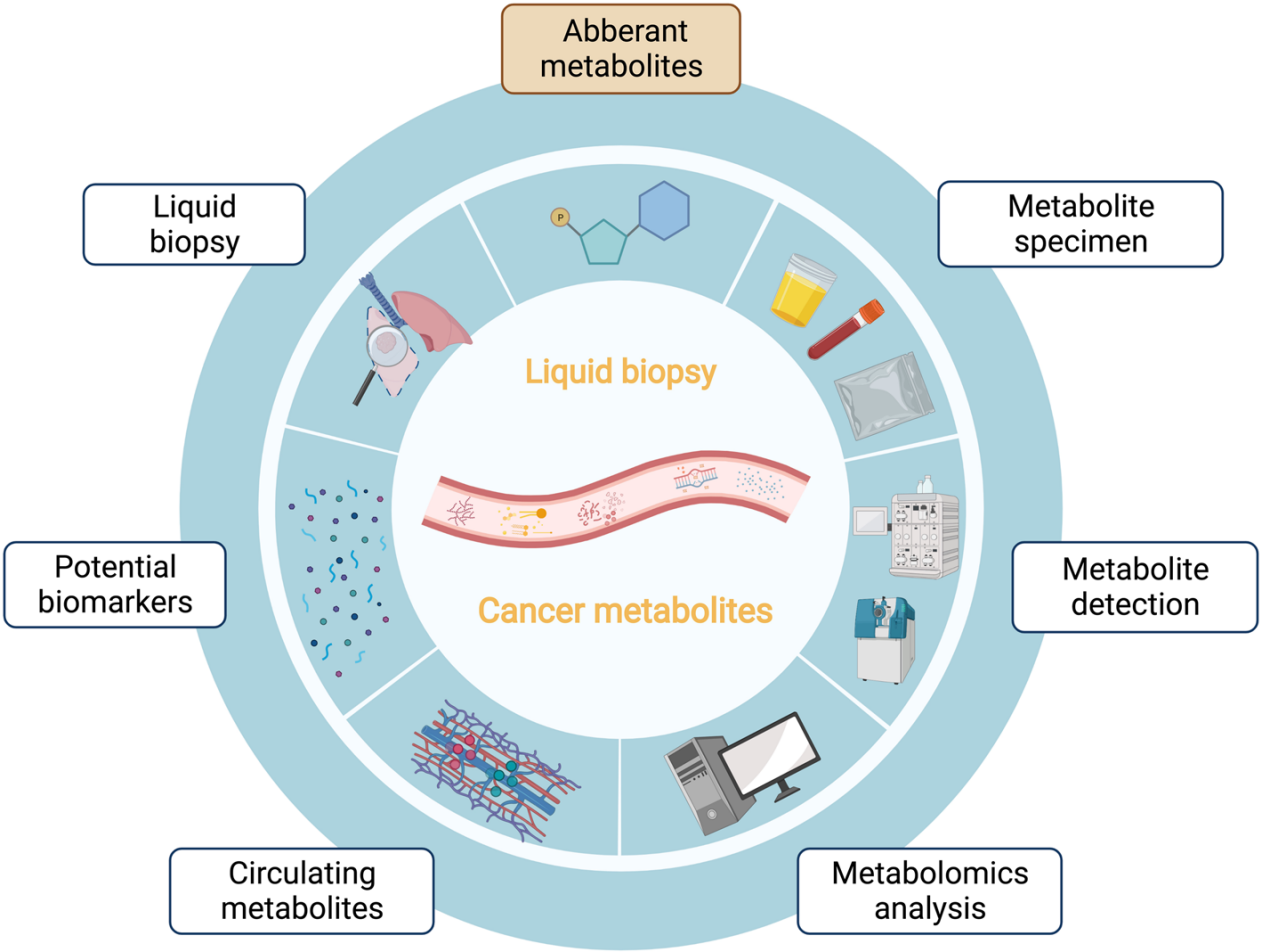
Overview of total contents. With the discovery of abnormal metabolites in cancer and the development of high-throughput metabolomics, circulating metabolites are expected to be potential biomarkers for early detection and diagnosis of cancer in the cotext of liquid biopsy

The search for tumor biomarkers using metabolomics is a promising direction, as changes in abnormal metabolites can reflect tumor biological activity. By linking human physiology and cancer biology through the detection and analysis of metabolites, cancer metabolic biomarkers can eventually be established and applied in clinical cancer screening, diagnosis and treatment (Fig. 2). The biggest challenges of metabolomics are the vast amounts of metabolic data and chemical complexity of metabolites. However, with advances in research and technology, we propose that the field of metabolomics will gradually mature and be utilized in various cancer diagnostics and prognostics. circulating metabolites can be used as rapid and noninvasive diagnostic or prognostic biomarkers to support clinical decisions and cancer management.

## Data Availability

No supporting data.

## Abbreviations

AA: Arachidonic acid
ANOVA: Analysis of variance
BCAA: Branched-chain amino acid
CE-MS: Capillary electrophoresis-mass
Cer: Ceramide
CRC: Colorectal cancer
CSC: Cancer stem cell
CTC: Circulating tumor cell
ctDNA: Circulating tumor DNA
DNN: Deep neural networks
EMT: Epithelial-mesenchymal transition
FDR: False-discovery rate
FFA: Free Fatty acid
FH: Fumarate hydratase
GC–MS: Gas chromatography-mass
GMSM: Gut microbiome-associated serum metabolite
HCC: Hepatocellular carcinoma
IDH: Isocitrate dehydrogenase
IL4I1: Interleukin-4-induced-1
Kyn: Kynurenine
LA: Linoleic acid
LC–MS: Liquid chromatography-mass
LPC: Lyso Phosphatidyl choline
LXR: Liver X receptor
m5C: 5-Methylcytosine
m6A: N6-methyladenosine
MALDI-TOF: Matrix-assisted laser desorption/ionization time of flight mass spectrometry
miRNA: Micro RNA
mRNA: Messenger RNA
MS: Mass spectrometry
mTORC1: Mechanistic target of rapamycin complex 1
NAM: Nicotinamide
NGS: Next-generation sequencing
NMR: Nuclear magnetic resonance
NSCLC: Non-small-cell lung cancer
O-PLS: Orthogonal partial least squares
OXPHOS: Oxidative phosphorylation
PC: Phosphatidylcholines,
PCA: Principal component analysis
PE: Phosphatidyl ethanolamine
PLS: Partial least squares
RCC: Renal cancer carcinoma
RF: Random forests
SAM: S-adenosyl methionine
SDH: Succinate dehydrogenase
SM: Sphingomyelin
SVM: Support vector machine
Trp: Tryptophan
TS: Thymidylate synthase
t-SNE: T-Distributed Stochastic Neighbor Embedding
VIP: Variable Importance in Projection
